# Evaluation of Susceptibility of the Human Pathogen *Helicobacter pylori* to the Antibiotic Capreomycin

**DOI:** 10.1155/2022/8924023

**Published:** 2022-07-31

**Authors:** Suriyan Sukati, Imran Sama-ae, Gerd Katzenmeier, Sueptrakool Wisessombat

**Affiliations:** ^1^Department of Medical Technology, School of Allied Health Sciences, Walailak University, Nakhon Si Thammarat 80160, Thailand; ^2^Hematology and Transfusion Science Research Center (HTSRC), Walailak University, Nakhon Si Thammarat 80160, Thailand; ^3^Akkhraratchakumari Veterinary College, Walailak University, Nakhon Si Thammarat 80160, Thailand; ^4^Center of Excellence Research for Melioidosis and Microorganisms (CERMM), Walailak University, Nakhon Si Thammarat 80160, Thailand

## Abstract

*Helicobacter pylori* infection causes gastritis, peptic ulcer disease, mucosa-associated lymphoid tissue lymphoma, and gastric cancer and can also promote thrombosis. It is estimated that approximately 4.5 billion individuals are infected, thus rendering *H. pylori* the most prevalent microbial pathogen. Currently established regimes for antibiotic treatment are massively challenged by increasing drug resistance and the development of novel antimicrobial therapies is urgently required. The antibiotic capreomycin is clinically used against multiple drug-resistant strains of *Mycobacterium tuberculosis*. It targets the complex between TlyA, a hemolysin- and RNA-binding protein, and the bacterial rRNA. In this study we have explored the possible antibacterial effects of capreomycin against several strains of *H. pylori* and found only moderate activity which was comparable to metronidazole-resistant strains. Molecular docking of capreomycin to TlyA proteins from *H. pylori* and *M. tuberculosis* identified several residues within TlyA which interact with the drug; however, binding affinities of *H. pylori*– TlyA for capreomycin appear to be higher than those of *Mycobacterium*– TlyA. The data suggest that capreomycin may warrant further investigations into its potential use as antibiotic against *H. pylori.*

## 1. Introduction

*Helicobacter pylori*, a spiral-shaped, Gram-negative bacterium, colonizes the mucous layer of the gastric epithelium and is the most prevalent microbial pathogen. It is estimated to infect half of the world's population [1], causing a range of severe gastropathies, such as peptic ulcer disease (PUD), chronic gastritis, and gastric adenocarcinoma [2]. Moreover, recent evidence suggests that *H. pylori* infection could promote atherothrombosis [3]. This renders *H. pylori* the only microbial pathogen known to cause cancer [4]. Eradication of *H. pylori* by antibiotic treatment regime is strongly recommended to decrease the incidence of gastric diseases [5]. However, resistance to commonly used antibiotics has been growing worldwide, frequently leading to eradication failure or unclear therapeutic outcomes [6]. The discovery of novel compounds is urgently required to improve current eradication therapies. A triple therapy using a proton-pump inhibitor (PPI, omeprazole, pantoprazole) and antibiotics, usually clarithromycin combined with either metronidazole or amoxicillin, has been employed for decades as first-line treatment [7]. Since resistance levels increased dramatically over the past few years, the triple therapy is now progressively replaced in areas of high resistance against clarithromycin and metronidazole by a bismuth-containing “three-in-one” quadruple therapy (BQT) [8]. A number of novel treatment options are currently subject to intensive investigation. These include curcumin derivatives [9], antimicrobial peptides (AMPs) [10], probiotics [11], and phytomedicine [12].

An interesting approach to the problem of drug resistance was recently addressed by Grande et al. [13]. The authors have shown that carbonic anhydrases, which are essential for a number of metabolic processes, could be selectively inhibited by carvacrol and thymol, thus resulting in impaired biofilm formation and release of outer membrane vesicles. The compounds demonstrated a high degree of selective toxicity against the pathogen when compared to probiotic microbial species of the gut. This finding would offer the prospect of an improved treatment strategy which possibly could work in combination with established antibiotic therapies.

Earlier studies had shown that capreomycin, an aminoglycoside antibiotic originally isolated from *Streptomyces capreolus*, and the similar compound viomycin could inhibit formation of the translation initiation complex and interfered with ribosomal protein biosynthesis by arresting the peptidyl-tRNA in the acceptor site [14, 15].

The ‘nonconventional' hemolysin TlyA was previously shown to confer sensitivity to capreomycin by modiﬁcation of nucleotides C1409 in helix 44 of 16S rRNA and C1920 in helix 69 of 23S rRNA (numbering corresponds to *E. coli* rRNA) [16, 17]. Bioinformatics analysis revealed that the TlyA protein sequence comprises a binding site for S-adenosyl methionine, a ribosomal protein binding domain (S4), and FtsJ-like motifs (encompassing residues 62–247) typical for RNA methyltransferases [18]. Mutations within the *tly*A gene from *M. tuberculosis* which inactivate the methyltransferase result in resistance to the antibiotic, while the introduction of wild-type *tly*A into *tly*A-negative mutants conversely restored sensitivity to capreomycin [19].

Capreomycin is widely used as ‘second-line' drug for the treatment of multidrug-resistant (MDR) *M. tuberculosis* and its mechanism of action and its benefit in clinical use are relatively well described [20, 21]. In light of its frequent use as potent antibiotic for the treatment of tuberculosis, it is surprising that, to the best of our knowledge, the effect of capreomycin on *H. pylori* has not been evaluated in detail thus far. TlyA from *H. pylori* displays a relatively high degree of homology to the corresponding protein from *M. tuberculosis*, thus suggesting that similar effects on activity could be detected for TlyA from *H. pylori* [14].

In the present report we sought to demonstrate antimicrobial activity of capreomycin against *H. pylori* and to characterize the effectiveness of the compound by comparison of its activity with those of amoxicillin, clarithromycin, metronidazole, and levofloxacin. At present, there is no crystallographic structure available for capreomycin bound to TlyA; however, 3-dimensional structures for the complex of capreomycin with rRNA have been obtained [22].

We have therefore attempted to gain further insight into the interaction of capreomycin with the TlyA-rRNA drug target by molecular modelling. Our data reveal that capreomycin demonstrates moderate antimicrobial activity against several strains of *H. pylori* and molecular models suggest the existence of a drug-TlyA cocomplex.

## 2. Materials and Methods

### 2.1. Bacterial Strains and Culture Conditions

Reference strains including *H. pylori* NCTC 11637 and *H. pylori* NCTC 11638 were cultured on Columbia blood agar base (Fluka, Switzerland) containing 10% defibrinated horse blood (Oxoid, UK). Plates were incubated at 37°C for 48 h under a microaerobic atmosphere using gas pack system (Mitsubishi, Japan). *H. pylori* strains MTCC-Hp01, MTCC-Hp02, and MTCC-Hp03 were obtained from the Medical Technology Culture Collection, Walailak University. Strains of *H. pylori* were verified by a *Campylobacter*-like organism (CLO) test (Kimberly-Clark, USA). The CLO test was performed according to the manufacturer's instructions, and the results were interpreted after 24 h. Isolated strains were analyzed with biochemical tests including catalase, oxidase, and urease.

### 2.2. Antibiotic Susceptibility Testing

The agar dilution method followed the protocol described by Clinical and Laboratory Standards Institute (CLSI) 2020 guidelines (M7-A5). Briefly, 2 *μ*L of bacterial suspension was inoculated at 0.5 McFarland turbidity concentration into Mueller-Hinton broth (Oxoid, UK), supplemented with 7% horse blood, 7% FCS, containing twofold dilutions of antibiotics and then incubated for 72 h under microaerophilic conditions (5% O_2_, 10% CO_2_, and 85% N_2_) using gas pack system (Mitsubishi, Japan) at 37°C. The concentrations of amoxicillin (AMX), capreomycin (CAP), clarithromycin (CLA), and levofloxacin (LVX) (Sigma Aldrich, Germany) ranged from 0.016 to 64 *μ*g/mL, while metronidazole (MTZ) (Sigma Aldrich, Germany) concentration ranged from 0.016 to 256 *μ*g/mL. Minimal Inhibitory Concentration (MIC) was defined as the lowest antibiotic concentration that completely inhibited visible growth of the bacteria.

### 2.3. Antibiotic Susceptibility Testing by E-Test

Susceptibility to amoxicillin, clarithromycin, metronidazole, and levofloxacin was tested by the E-test, following manufacturer's instructions (AB BIODISK, Sweden). Antimicrobial concentration ranged from 0.015 to 256 *μ*g/mL. The bacteria were cultured for 48 h in blood-supplemented Columbia agar and a bacterial suspension adjusted to 10^7^ CFU/mL was inoculated directly onto Mueller-Hinton agar supplemented with 5% sheep blood obtained commercially (bioMerieux, France). E-test was applied to the culture media within 30 min of inoculation. Plates containing the E-test were incubated under a microaerobic atmosphere. After 72 h of incubation, the MIC was determined by determining the point where the growth curve coincided with the scale number on the E-test strip.

### 2.4. The Structure Prediction for H. *pylori* Methyltransferase TlyA by Molecular Modelling

The structural interaction between capreomycin and TlyA was investigated using computational modelling methods. TlyA represents the 16S/23S rRNA (Cytidine-2′-O)-methyltransferase protein of *H. pylori* (Hp TlyA). Due to the absence of a crystal structure for this protein, the SWISS-MODEL was used to predict the three-dimensional structure of this protein [23]. The FASTA sequence of TlyA was retrieved from UniProt (https://www.uniprot.org/) (UniProt ID : A0A402DXR) and used as target sequence for homology modelling. In addition, TlyA of *Mycobacterium tuberculosis* (MtbTlyA, PDB ID : 5KS2) was selected as a template for modelling. Finally, the predicted 3D structure was evaluated using VERIFY 3D to determine the compatibility of the atomic model (3D) with its own amino acid sequence (1D) by assigning structural classes based on location and environment (alpha, beta, loop, polar, nonpolar, etc.) and comparison of the results to established structures [24].

### 2.5. Preparation of Protein and Ligand Structures for Molecular Docking

We have used molecular docking to measure the binding energies of capreomycin to Hp TlyA protein. The binding energies of Hp TlyA were compared to those for the MtbTlyA, the drug target for capreomycin in tuberculosis treatment [16, 25, 26]. Prior to docking, the protein structures were dewatered to expose only the amino acid residues. Polar hydrogens were assigned, nonpolar hydrogens were merged, and Kollman charges were added (Reference DOI: https://doi.org/10.1016/j.imu.2020.100331). Finally, partial charges and atom types were assigned to stabilize the protein and were saved in the Protein Data Bank (PDB), using partial charge (Q) and atom type (*T*) (PDBQT) formats. The PubChem database was searched for available structures of capreomycin (PubChem CID: 3000502). Energy minimization was performed to obtain 3D structures with proper bond lengths between different atoms using open label option in PyRx 0.8 [25, 27], and Universal Force Field (uff), a full periodic table force field for molecular mechanics and molecular dynamics simulations, was selected [28]. The structures were saved in PDBQT format using PyRx version 0.8 [25]. Finally, polar hydrogens and Gasteiger charges were introduced, and nonpolar hydrogens were merged using the AutoDock Auxiliary Tool (ADT) version 4.2 [29, 30].

### 2.6. Molecular Docking Simulation

Molecular docking was performed using AutoDock4 version 4.2 [29, 30]. The Lamarckian Genetic Algorithm was used in order to combine global search (genetic algorithm alone) and local search (Solis and Wets algorithm) [30]. Each docking step consisted of an initial population of 250 randomly placed individuals, a maximum number of 25 million energy evaluations, a mutation rate of 0.02, a crossover rate of 0.80, and an elitism value of 1.0 (https://www.nature.com/articles/s41598-021-83474-9?proof=tr). For the local search, the so-called pseudo-Solis and Wets algorithm was applied using a maximum of 250 iterations per local search. 250 independent docking runs were carried out for each ligand. The grid maps representing the system in the actual docking process were calculated with AutoGrid. The dimension of the grid was set to sufficiently cover the active site (126 × 126 × 126 Å), with a spacing of 0.486 Å. The protein-ligand lowest binding energy (ΔGbind) and the inhibitor constant (*K*_*i*_) were determined using AutoDock Auxiliary Tool (ADT) version 4.2 [28, 29].

### 2.7. Protein and Ligand Visualization

The proteins and ligands were visualized using BIOVIA Discovery Studio software [31], Protein Imager [32], and Mol^*∗*^ [33].

### 2.8. Molecular Dynamics Simulations of the Capreomycin-HpTlyA Complex

MD simulations were performed using the Desmond module from Schrödinger suite [34]. Hydrogen bonds were assigned using standard protocols. The protein and ligand complexes were then placed in the OPLS force field. After immersing the complex in a TIP3P water model and maintaining a distance of 10 Å from the center of the box, the energy minimization of the complexes was performed. Then, sodium and chloride ions were added to mimic the *in vivo* environment and neutralize the system. At 310.15 K and a pressure of approximately 1.01325 bar, molecular dynamic simulations were carried out using ensembles of constant numbers of particles, pressure, and temperature (NPT) for 100 ns with a recording interval of 100 ps [35]. The root mean square deviation (RMSD) trajectories of the protein-ligand interaction were calculated using the following formula:

(1) $$\begin{array}{c} \text{RMSD}x=\sqrt{\frac{1}{N}\sum_{i=1}^{N}\left(r_{i}^{\prime }\left(t_{x}\right)\right)-{\left(r_{i}\left(t_{ref}\right)\right)}^{2}}, \end{array}$$

where N is the number of selected atoms; *r*′ is the position of these selected atoms in frame *x* after overlapping in the reference frame, where the frame *x* is recorded at time *t*_*x*_; and *t*_*ref*_ is the reference time. A new iteration of this process was performed for each subsequent frame of the simulation [34].

The root mean square fluctuation (RMSF) trajectories of the protein residues were calculated using the formula:

(2) $$\begin{array}{c} \text{RMSF}i=\sqrt{\frac{1}{T}\sum_{t=1}^{T}\langle \left(r_{i}^{\prime }\left(t\right)\right)-{\left(r_{i}\left(t_{ref}\right)\right)}^{2}\rangle }, \end{array}$$

where *T* denotes the trajectory time interval over which the RMSF is calculated; *r*′ denotes the position of atoms in the residue I after the superposition in the reference; *r*_*i*_ denotes the position of residue I; and *t*_*ref*_ denotes the reference time; and the angle brackets denote that the square distance is averaged on the residue selection of atoms [34].

The root mean square fluctuation (RMSF) trajectories of the ligand atoms were calculated using the formula:

(3) $$\begin{array}{c} \text{RMSF}i=\sqrt{\frac{1}{T}\sum_{t=1}^{T}\left(r_{i}^{\prime }\left(t\right)\right)-{\left(r_{i}\left(t_{ref}\right)\right)}^{2}}, \end{array}$$

where *T* denotes the trajectory time interval over which the RMSF is calculated; *t*_*ref*_ denotes the reference time; *r* denotes the position of atom I in the reference at time *t*_*ref*_; and *r*′ denotes the position of atom I in the reference at time *t* after superposition on the reference frame [34].

The protein and ligand RMSD, protein and ligand RMSF, and protein-ligand contacts were analyzed using the simulation interaction diagram tool of Desmond Schrödinger's module [34, 35].

## 3. Results and Discussion

Our objective was to evaluate possible antimicrobial effects of the aminoglycoside antibiotic capreomycin against the gastric pathogen *H. pylori*. The presence of the TlyA protein in both species of microorganisms, *Mycobacterium tuberculosis* and *Helicobacter pylori*, could offer the prospect of a novel treatment option for drug-resistant strains of *H. pylori*.

Results of antimicrobial susceptibility tests with both reference strains and clinical isolates are shown in Table 1. Agar dilution revealed that AMX, CAP, CLA, MTZ, and LEV had activity against all strains of *H. pylori* with MIC values of 0.064–2 *μ*g/mL, 0.25–64 *μ*g/mL, 0.25–4 *μ*g/mL, 2–64 *μ*g/mL, and 0.015–8 *μ*g/mL, respectively. MIC values as determined by the E-test for AMX, CLA, MTZ, and LEV were 0.125–16 *μ*g/mL, 0.25–2 *μ*g/mL, 2–64 *μ*g/mL, and 0.015–8 *μ*g/mL, respectively. The prevalence of antibiotic resistance among 5 isolates was 20% for metronidazole and levofloxacin, while no resistant strains were found for amoxicillin, capreomycin, and clarithromycin (0%). According to European Committee on Antimicrobial Susceptibility Testing (EUCAST), resistance is defined by these MIC breakpoints: MIC >0.12 *μ*g/mL for AMX, >0.5 *μ*g/mL for CLA, >8 *μ*g/mL for MTZ, and >1 *μ*g/mL for LEV [36].

The CLSI E-test is considered the most popular because of its flexibility in a routine laboratory. It should be noted, however, that for drug discovery against *M. tuberculosis*, screening methods based on high-throughput, absolute concentration methods have been employed [37].

Contrary to our expectations, capreomycin demonstrated a relatively moderate antimicrobial activity with MIC values comparable to those of metronidazole-resistant strains. While we have confirmed that the antibiotic has an inhibitory effect on all strains of *H. pylori*, the reasons for the fair activity are not entirely clear at present. A recent *in vitro* study on *M. tuberculosis* has determined a median MIC of capreomycin to 1 mg/L, with 62.8% of the strains being above the WHO breakpoint of 2.5 mg/L [38].

It is conceivable that large variations in susceptibility exist among strains and clinical isolates of *H. pylori*. Formally, we cannot rule out the inclusion of capreomycin-resistant strains in our study as we have also found a noticeable proportion of strains resistant to metronidazole and levofloxacin. In particular, resistance to metronidazole has increased over the past years, while amoxicillin and levofloxacin show generally lower rates of resistance [39–41]. We concede that screening a larger number of drug-sensitive strains would be required to assess the levels of resistance to capreomycin, especially in cases where previous antibiotic therapy was applied. Determination of genotypic differences between sensitive and resistant strains would allow for a better discrimination against resistant strains which may affect data for susceptibility. However, at present there is little information on resistance mechanisms operating against aminoglycoside antibiotics in *H. pylori*. In *M. tuberculosis*, low-level resistance to aminoglycosides is attained by the Eis acetyltransferase which inactivates the antibiotics [42].

In general, aminoglycoside antibiotics are not considered a promising treatment option for *H. pylori*, mainly because of their problematic pharmacological properties such as poor gastric membrane permeability which necessitates parenteral administration. In addition, their pH-dependent activity may render them inactive in the acidic stomach environment [43].

Capreomycin is interestingly classified as bacteriostatic rather than bactericidal (Capreomycin, LiverTox, NCBI Bookshelf (https://nih.gov). It is currently used only as a secondary agent for the treatment of multidrug-resistant mycobacterial infections in combination with isoniazid, ethambutol, pyrazinamide, and/or rifampin (but not streptomycin or other aminoglycosides). Capreomycin displays side effects typical for most aminoglycoside antibiotics such as tinnitus and ototoxicity, renal dysfunction, and injection site irritation. It seems surprising that even despite its frequent use for the treatment of tuberculosis, the pharmacological properties of capreomycin such as protein binding, metabolism, and excretion are not well characterized at present.

**Figure 1:**
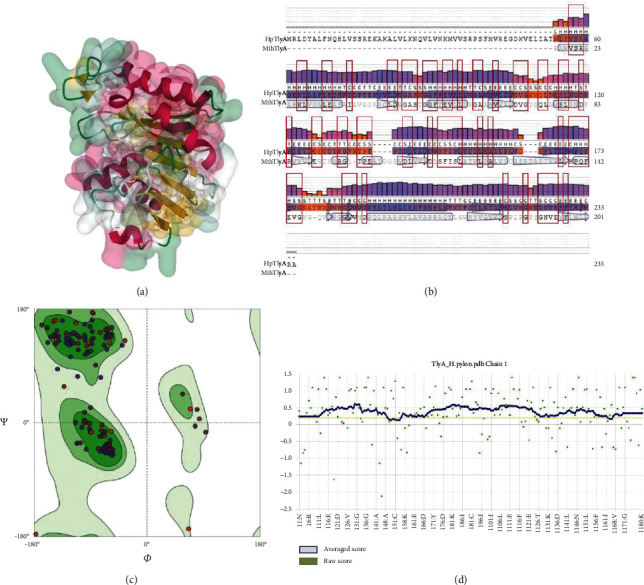
The predicted three-dimensional structures of HpTlyA. (a) 3D structure of HpTlyA. (b) Residue quality. (c) Ramachandran Plots. (d) 3D verification by VERIFY3D.

3D structural models of HpTlyA were constructed by SWISS-MODEL server using MtbTlyA as a template. The 3D structure model of HpTlyA is illustrated in Figure 1(a). The percentage of sequence identity with the template was 37.08% (Figure 1(b)). The Ramachandran plot of the HpTlyA model identified 93.26% of the residues in most favored regions with 0.00% of the residues in outlier regions, thus indicating a good stereochemical quality of the obtained protein structures (Figure 1(c)). The QMEANDisCo Global of the predicted structure was 0.68 ± 0.06. Analysis by VERIFY3D showed that 95.56% of residues had an average 3D-1D score ≥0.2, indicating an acceptable compatibility of the atomic model (3D) with its own amino acid sequence (Figure 1(d)).

**Figure 2:**
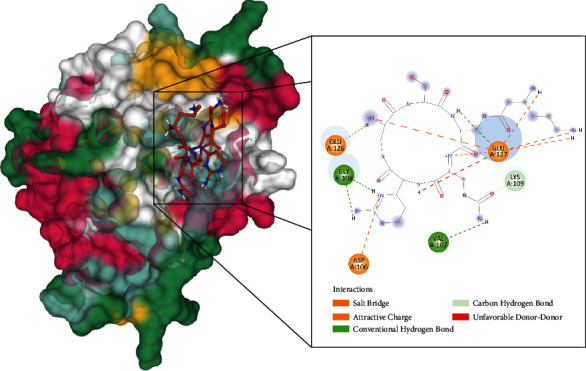
The interaction of capreomycin toward the HpTlyA protein predicted by molecular docking.

**Figure 3:**
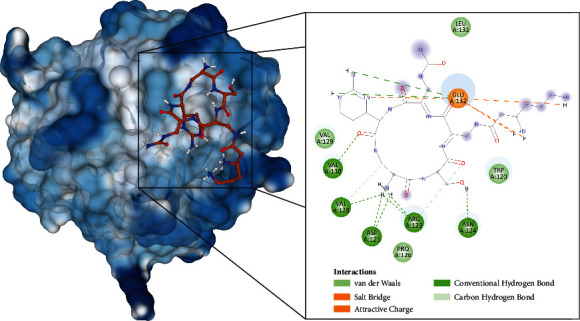
The interaction of capreomycin toward the MtbTlyA protein predicted by molecular docking.

Molecular docking of capreomycin to the HpTlyA and MtbTlyA proteins was performed using AutoDock 4. The results are presented in Figures 2 and 3 and Table 2. Capreomycin demonstrated a binding potential towards HpTlyA with ΔGbind of -7.43 kcal/mol, and the inhibitory constant (*K*_*i*_) of 3.56 *μ*M. The compound interacts with residues Asp106, Glu126, and Glu127 through salt bridges, and with residues Val107, Gly108, Lys109, Glu126, and Glu127 through hydrogen bond (Figure 2). Capreomycin demonstrated a binding potential towards HpTlyA with binding energy (ΔGbind) of -6.27 kcal/mol and the inhibitor constant (*K*_*i*_) of 25.3 *μ*M. The compound interacts with residues Trp120, Pro126, Val128, and Leu131 through van der Waals and with residues Arg123, Asn124, Asp125, Val128, Val130, and Glu132 through hydrogen bonds. It forms a salt bridge with Glu132 (Figure 3). Capreomycin binds to HpTlyA at the same positions where it binds to MtbTlyA. The binding energy of capreomycin to HpTlyA was lower than that of capreomycin bound to MtbTlyA. This would indicate that the binding affinity of capreomycin to the HpTlyA is higher than the binding affinity of capreomycin to its drug target, MtbTlyA.

Crystal structures of the 70S ribosome in complex with tRNAs, viomycin, and capreomycin were resolved at 3.3–3.5 Å resolution, respectively [22]. Noteworthy, both antibiotics bind to the interface between the ribosomal subunits formed by helix 44 of the small subunit and helix 69 of the large subunit. The structures suggest that the two tuberactinomycins function as inhibitor of translocation by arresting the tRNA in the A-site in a pretranslocation conformation.

A recent study has investigated binding energies of capreomycin and streptomycin in complex with the tuberculosis bacterial ribosome subunits using density functional theory (DFT) and the molecular fractionation with conjugated caps (MFCC) approach [44]. For capreomycin bound to the 30S and 50S ribosomal subunits, contributions of hydrogen bonds and hydrophobic interactions in the drug-target complex were characterized and residues within capreomycin contributing to target binding were identified.

A remarkable finding of this investigation was that long-range distances of drug binding to the nucleosides of rRNA are an important factor for the activity of capreomycin and streptomycin. It was found that drug residues with lower binding energy values are separated between 6 and 11 Å in the 30S subunit and between 26 and 30 Å in the 50S subunit from the ribosome. The predominant interaction of capreomycin with the ribosome occurs in the 30S subunit through hydrogen bonds at nucleotides A1493 (29.69 kcal/mol), G1494 (26.94 kcal/mol), G1491 (19.27 kcal/mol), A1492 (14.75 kcal/mol), and A1408 (12.83 kcal/mol) with the binding energies indicated in parentheses.

The TlyA methyltransferase catalyzes the methylation of nucleotides C1920 and C1409 conducive to an increased susceptibility to capreomycin, or *vice versa*; failure to methylate these residues through absence of the *tly*A wild-type gene confers higher resistance to the drug. Mutations in the rRNA (A1401G, C1402T, and G1484T) prevent the fixation of TlyA to the RNA, thus rendering the ribosome resistant to drugs. Capreomycin and streptomycin share 17 conserved residues and consequently exhibit very similar mechanisms of action. Nevertheless, energetic contributions of individual residues to drug binding appear to modulate the effect of the antibiotics and result in different pharmacological activity spectra.

While the report of Vianna et al. has identified the most relevant energetic interactions between capreomycin and the ribosomal RNA, our data support the possibility of a complex formation between the drug and TlyA. Preliminary data suggest the existence of a supramolecular complex of streptomycin with the ribosomal protein S12 formed by amino acid residues with high binding energy values [44].

**Figure 4:**
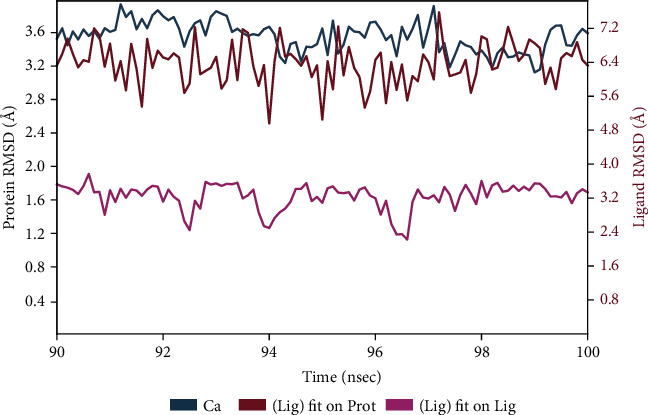
Plot of protein-ligand RMSD.

**Figure 5:**
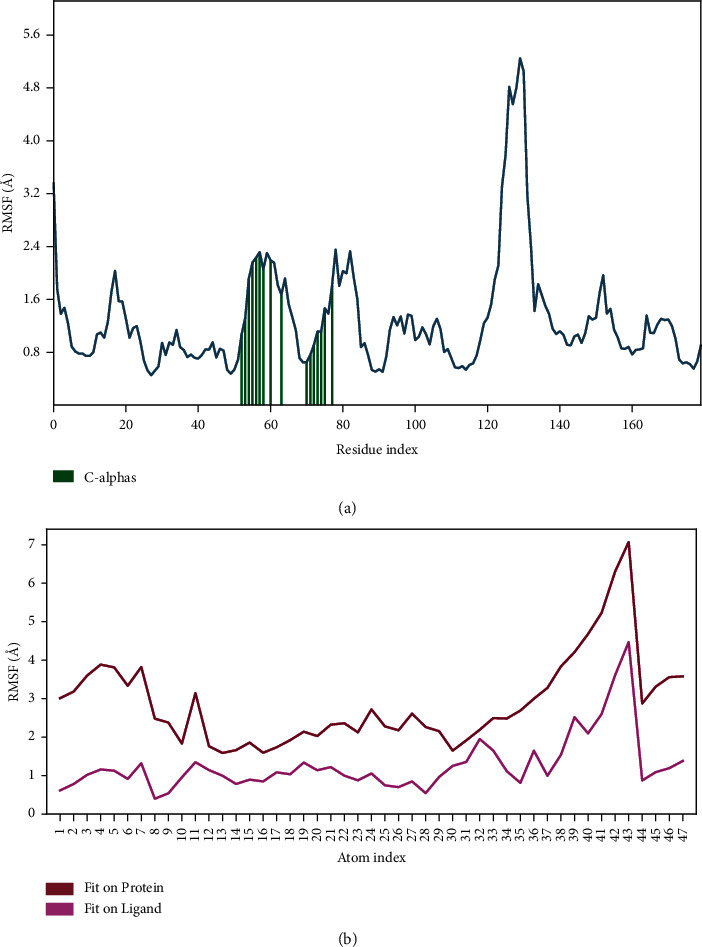
Plot of protein-ligand RMSF. (a) Protein RMSF. (b) Ligand RMSF.

**Figure 6:**
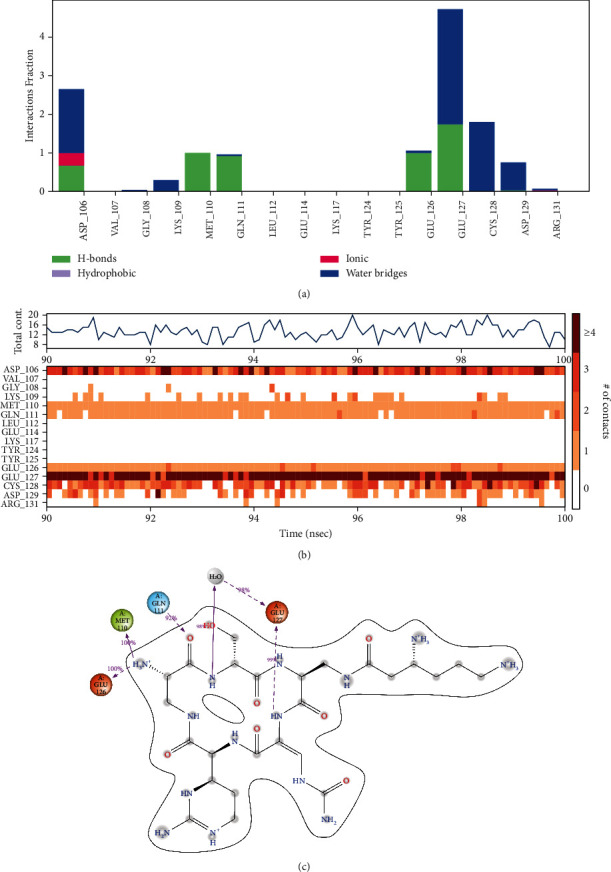
Protein-ligand contacts. (a) Histogram of protein-ligand contacts categorized by type of interactions: hydrogen bonds (green), hydrophobic interactions (purple), ionic bonds (magenta), and water bridges (blue). (b) Timeline representation of the interactions throughout 90–100 ns. (c) A schematic representation of the detailed interactions of the ligand atoms with the protein residues.

The dynamic motions of the docked complexes and the binding stabilities were further analyzed by molecular dynamic simulations at 100 ns using the Desmond module of Schrödinger's suite [34]. The results of MD simulations of the capreomycin-HpTlyA complex are shown in Figures 4–6.

The root mean square deviation (RMSD) quantifies the average change in displacement of a selection of atoms relative to a reference frame for a particular frame. For the Protein RMSD (P-RMSD), these plots illustrate the RMSD evolution of a protein (left *Y*-axis). The P-RMSD is calculated based on the atom selection after aligning all protein frames with the reference frame backbone. During the simulation, the monitoring of the P-RMSD can provide insight into its structural conformation. For the ligand RMSD (L-RMSD), the L-RMSD value (right *Y*-axis) indicates the stability of the ligand in relation to the ligand (aligned on ligand) and protein (aligned on protein). The result demonstrated that the P-RMSD values of the capreomycin-HpTlyA complex were stable at around 90–100 ns, the highest value was 3.94 Å, and the lowest value was about 3.12 Å, indicating that the system has equilibrated during this simulation. The L-RMSD values (aligned on ligand) of the capreomycin-HpTlyA complex show the highest value was 3.77 Å, and the lowest value was 2.22 Å. The L-RMSD values (aligned on protein) of the capreomycin-HpTlyA complex show the highest value was 7.58 Å, and the lowest value was 4.96 Å (Figure 4).

The root mean square fluctuation (RMSF) can characterize local changes in the protein chain and the positions of the ligand atoms. For the protein RMSF (P-RMSF), the peaks in this plot correspond to the protein regions that fluctuate the most during the simulation. The P-RMSF of the capreomycin-HpTlyA complex strongly fluctuated at amino acid residues Ala178, Thr179, Lys180, Arg181, Asn182, Lys183, Lys184, and Gly185. These residues were not ligand contacts residues (Figure 5(a)). Ligand RMSF (L-RMSF) shows the fluctuations of the ligand broken down by atom. L-RMSF elucidates the interaction of ligand fragments with the protein and their entropic role in the binding event. The ‘Fit Ligand on Ligand' line in the graph depicts the ligand fluctuations with respect to the ligand. While the ‘Fit Ligand on Protein' line in the graph depicts the ligand fluctuations with respect to the protein. After aligning the protein-ligand complex on the protein backbone, the L-RMSF on the ligand heavy atoms is determined. The fluctuations for L-RMSF (relative to the ligand) and L-RMSF (relative to the protein) of the capreomycin-HpTlyA complex were found at intervals of around 0.40–4.47 Å and 1.59–7.07 Å, respectively. The most fluctuated atom was atom 43, NH_3_^+^ (Figure 5(b)).

During the simulation, the interactions of the protein with the ligand can be monitored. The protein-ligand contacts diagrams for the capreomycin-HpTlyA complex were illustrated in Figure 6. The stacked bar charts demonstrated that the complexes exhibited H-bonds, hydrophobic interactions, ionic bonds, and water bridges during the simulation (Figure 6(a)). A timeline representation of the interactions and contacts showed that the number of specific contacts made by protein with the ligand during the trajectory (90.00 to 100.00 ns) was in the range of 8–20 contacts. Additionally, the results demonstrated that Asp106, Met110, Gln111, Glu126, Glu127, and Cys128 were the protein's residues that frequently interacted with the ligands (Figure 6(b)). A schematic of the detailed ligand atom interactions with these protein residues was illustrated in Figure 6(c).

The identification of binding sites on the ribosome may facilitate the development of drugs with improved pharmacological features and efficacy against drug-resistant strains. It is, however, still an open question as of whether and to what extent these observations can be extrapolated to *H. pylori*.

Despite their obvious limitations, aminoglycosides may nevertheless constitute a novel and promising option for antibiotic therapy against *H. pylori*, as improved delivery systems such as gentamicin-intercalated smectite hybrids with optimized pharmacological properties are currently being developed [45].

## 4. Conclusions

In light of these developments, we believe that we have provided additional evidence for a conceivable role of capreomycin for the treatment of *H. pylori* diseases. Capreomycin may warrant further investigation, in particular an analysis of PK/PD (pharmacokinetic and pharmacodynamic parameters), as potential anti – *H. pylori* drug.

## Figures and Tables

**Table 1:** Antimicrobial susceptibility testing of *H. pylori* by agar dilution and E-test.

	EUCAST breakpoints (*μ*g/mL)	MIC (*μ*g/mL)		Resistance rate (%)^*a*^	
		Agar dilution	E-Test	Agar dilution	E-Test
Amoxicillin (AMX)	>0.12	0.064–2	0.125–16	0	0
Capreomycin (CAP)	NA	0.25–64	NA	NA	NA
Clarithromycin (CLA)	>0.5	0.25–4	0.25–2	0	0
Metronidazole (MTZ)	>8	2–64	2–64	20	20
Levofloxacin (LEV)	>1	0.015–8	0.015–8	20	20

^*a*^H. *pylori* strains MIC was greater than the breakpoint concentration. NA = nonapplicable.

**Table 2:** Binding affinity and inhibitory constant prediction of capreomycin against HpTlyA and MtbTlyA.

Compounds	PubChem CID	Binding affinity (kcal/mol)	Inhibitory constant	Interactive residues	Interactive bond (s)
*H. pylori*'s 16S/23S rRNA (Cytidine-2′-O)-methyltransferase TlyA (HpTlyA)					
Capreomycin	3000502	−7.43	3.56 uM	Asp106	Salt bridge
				Val107	Conventional hydrogen bond
				Gly108	Conventional hydrogen bond
				Lys109	Carbon hydrogen bond
				Glu126	Salt bridge
				Glu127	Conventional hydrogen bond
					Salt bridge

*M. tuberculosis*'s 16S/23S rRNA (Cytidine-2′-O)-methyltransferase TlyA (MtbTlyA)					
Capreomycin	3000502	−6.27	25.3 uM	Trp120	van der Waals
				Arg123	Conventional hydrogen bond
					Carbon hydrogen bond
				Asn124	Conventional hydrogen bond
				Asp125	Conventional hydrogen bond
				Pro126	van der Waals
				Val128	Conventional hydrogen bond
					Carbon hydrogen bond
				Val129	van der Waals
				Val130	Conventional hydrogen bond
				Leu131	van der Waals
				Glu132	Conventional hydrogen bond
					Salt bridge

## Data Availability

The data used to support this study are available from the corresponding author upon request.
